# Mutational screening of 10 genes in Chinese patients with microphthalmia and/or coloboma

**Published:** 2009-12-27

**Authors:** Xiaohui Zhang, Shiqiang Li, Xueshan Xiao, Xiaoyun Jia, Panfeng Wang, Huangxuan Shen, Xiangming Guo, Qingjiong Zhang

**Affiliations:** State Key Laboratory of Ophthalmology, Zhongshan Ophthalmic Center, Sun Yat-sen University, Guangzhou, China

## Abstract

**Purpose:**

To screen ten genes for mutations in 32 Chinese patients with microphthalmia and/or coloboma.

**Methods:**

Genomic DNA was prepared from 32 unrelated patients with microphthalmia (nine probands) and uveal coloboma (23 probands). Cycle sequencing was used to detect sequence variations in ten genes, including *BMP4*, *VSX2*, *CRYBA4*, *GDF6*, *OTX2*, *RAX*, *SIX3*, *SIX6*, *SOX2*, and *LRP6*. Variations were further evaluated in 96 unrelated controls by using restriction fragment length polymorphism (RFLP) or heteroduplex-single strand conformation polymorphism (HA-SSCP) analysis.

**Results:**

In the ten genes, a novel c.751C>T (p.H251Y) in *BMP4* was detected in a patient with bilateral microphthalmia and unilateral cataract. The c.751C>T variation is also present in his healthy brother (and possibly one of the normal parents). In addition, a novel c.608G>A (p.R203Q) in *SIX6* was identified in an internal control for optimizing experimental conditions. The internal control was from a girl with typical aniridia and an identified c.718C>T (p.R240X) mutation in *PAX6*, suggesting the c.608G>A variation in *SIX6* was unlikely to play a role in her ocular phenotype. The c.751C>T in *BMP4* and the c.608G>A in *SIX6* were not present in the 96 normal controls. In addition, 16 nucleotide substitutions, including eight known SNPs and eight new synonymous changes, were detected.

**Conclusions:**

Although the genetic etiology for microphthalmia and/or coloboma is still elusive, rare variations in the related genes, such as c.608 G>A in *SIX6* and c.751C>T in *BMP4*, may not be causative. These results further emphasize the importance of careful clinical and genetic analysis in making mutation-disease associations.

## Introduction

Microphthalmia and coloboma are important cause of congenital blindness, with a prevalence at 1.9–3.5/10,000 live births [1,2]. Microphthalmia and coloboma may be isolated or syndromic with the extraocular phenotype in one or both eyes. The disease exhibits diverse patterns of genetic inheritance, and the severity is variable, due to the genetic heterogeneity of the ocular malformation [3-8]. Mutations in several genes have been reported in patients with microphthalmia and/or coloboma [3,9-14], including *BMP4* (OMIM 112262), *VSX2* (*CHX10*; OMIM 142993), *CRYBA4* (OMIM 123631), *OTX2* (OMIM 600037), *RAX* (OMIM 601881), *SIX6* (OMIM 606326), and *SOX2* (OMIM 184429). Of these, mutations in *SOX2* account for about 10% of microphthalmia, anophthalmia, and coloboma [3,15,16]. However, mutations in *VSX2*, *CRYBA4*, *OTX2*, and *RAX* have been detected in about 2%–3% patients with microphthalmia, anophthalmia, and coloboma [10,11,15,17,18]. Mutations in *SIX3* and *GDF6* have been identified only in a few cases [19,20]. Recently, mutations in *BMP4* mutations have been detected in patients with anophthalmia-microphthalmia [9]. In addition, knockout of LRP6 in mice resulted in microphthalmia and coloboma, but has not yet been reported in humans [21].

Because most of these genes were usually studied individually, and mutation analysis of Chinese patients is rare so far, we screened 32 unrelated patients with microphthalmia and/or coloboma for mutations in ten related genes, including *BMP4*, *VSX2*, *CRYBA4*, *GDF6*, *OTX2*, *RAX*, *SIX3*, *SIX6*, *SOX2*, and *LRP6*, through sequencing analysis of the coding and adjacent intronic regions of the ten genes.

## Methods

### Patients and controls

**Figure 1:**
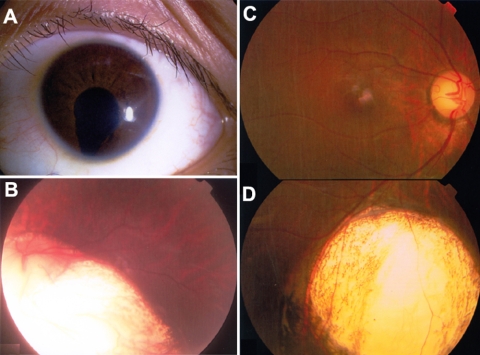
Uveal coloboma. **A** and **B** demonstrate iris coloboma (**A**) and choroid coloboma involving the optic disc (**B**). **C** and **D** show inferior choroid coloboma. (**D** did not align well with **C**).

**Figure 2:**
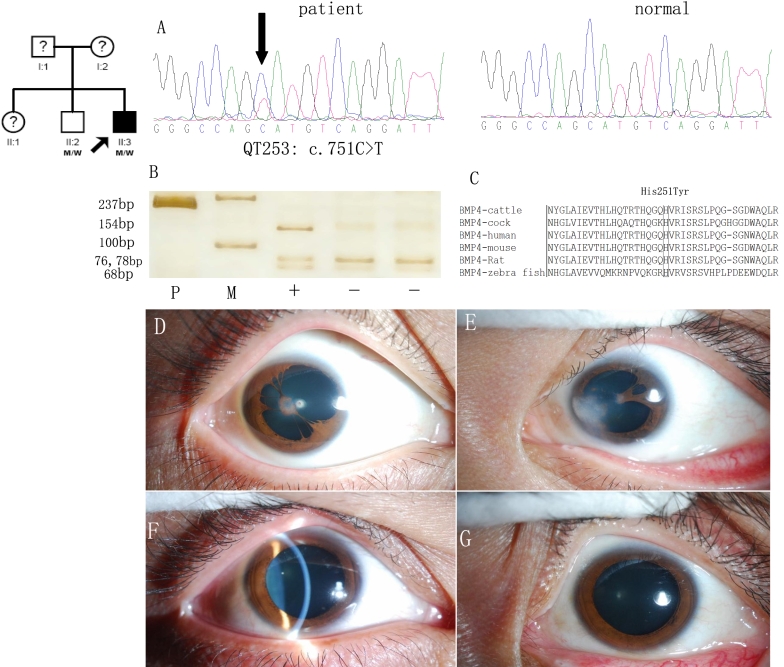
*BMP4* variation and associated phenotype. **A**: Sequence chromatogram demonstrated the c.751C>T variation in *BMP4* from the patient (left) and normal sequence from a control (right). **B**: The c.751C>T variation in *BMP4* detected by PCR-RFLP analysis (P: PCR products [237 bp] and M: marker, showing 100 bp and 250 bp, respectively; the plus sign [+] indicates CviAII-digested-products with heterozygous c.751C>T variation; the minus sign [-] indicates CviAII-digested products without the c.751C>T variation). **C**: Protein alignment of human BMP4 (residues 231–271) with other BMP4 orthologs from cattle, cock, mouse, rat, and zebra fish. **D** and **E**: Ocular phenotype of the proband showing bilateral microcornea, corneal opacities, multiple pupils, persistent iris membrane adhering to cornea (right eye) or lens capsule (left eye), and anterior pole cataract (right eye). **F** and **G**: Normal ocular phenotype of the proband’s healthy brother, which also carried the heterozygous c.751C>T variation in *BMP4*.

Thirty-two unrelated patients were recruited from our Pediatric and Genetic Eye Clinic, Zhongshan Ophthalmic Center. Clinical diagnoses of the 32 patients were microphthalmia (nine cases) and uveal coloboma (23 cases). Diagnosis of microphthalmia was based on criteria previously described [15], that is, a corneal diameter less than 10 mm and an axial length less than 20 mm. Of the nine cases with microphthalmia, eight met the criteria but one did not, although a small cornea and short axial length were recorded (details in results). On the other hand, the inclusion criteria for uveal coloboma were 1) congenital cleft in the inferior part of the iris and/or choroid (Figure 1) and 2) exclusion of aniridia or macular uveal coloboma. Besides this, an internal control sample for optimizing PCR and sequencing conditions was from a girl with aniridia and an identified PAX6 mutation. Furthermore, 96 unrelated controls were collected from normal volunteers. A previously established procedure was used for collecting subjects and obtaining informed consent [22]. This study was approved by the Institutional Review Board of Zhongshan Ophthalmic Center, China. Genomic DNA was prepared from venous blood from each participating individual [23].

### Mutation detection

PCR was used to amplify the coding exons and adjacent intronic sequences of the ten genes. Reference sequences for the ten genes are listed in Table 1. The primer sequences used to amplify the coding exons of the ten genes are listed in Table 2. The PCR products from individual exons from each individual were sequenced with the ABI BigDye Terminator cycle sequencing kit v3.1 (Applied Biosystems, Foster City, CA) and an ABI 3100 Genetic Analyzer (Applied Biosystems). Sequencing results and consensus sequences from the NCBI human genome database were compared by using the SeqManII program of the Lasergene package (DNAstar Inc., Madison, WI). Each mutation was confirmed by bidirectional sequencing. Mutation description followed the nomenclature recommended by the Human Genomic Variation Society.

**Table 1:** Genomic information of the 10 genes referred in this study.

Gene	Genomic DNA	mRNA	Protein
*BMP4*	NC_000014.8	NM_001202.3	NP_001193.2
*VSX2*	NC_000014.8	NM_182894.2	NP_878314.1
*CRYBA4*	NC_000022.10	NM_001886.2	NP_001877.1
*GDF6*	NC_000008.10	NM_001001557.1	NP_001001557.1
*OTX2*	NC_000014.8	NM_021728.2	NP_068374.1
*RAX*	NC_000018.9	NM_013435.2	NP_038463.2
*SIX3*	NC_000002.11	NM_005413.2	NP_005404.1
*SIX6*	NC_000014.8	NM_007374.2	NP_031400.2
*SOX2*	NC_000003.11	NM_003106.2	NP_003097.1
*LRP6*	NC_000012.11	NM_002336.2	NP_002327.2

The genomic DNA information was based on NCBI human genome build 36.3.

**Table 2:** Primers used for polymerase chain reaction amplification and sequencing of *BMP4*, *VSX2*, *CRYBA4*, *GDF6*, *OTX2*, *RAX*, *SIX3*, *SIX6*, *SOX2*, and  *LRP6*.

**Gene**	**Exon**	**Primer sequence (5'-3')**	**Amplicon size (bp)**	**Annealing temperature (°C)**
*BMP4*	3-F	CCATCTTGCCCCTCCATTTCTA	570	65
	3-R	CTTCTTCCCCAGGGCTTTCACT		
	4a-F	TGCTTATTTTCCCCCAGTAGGT	704	62
	4a-R	GGCGCCGGCAGTTCTTATTCTT		
	4b-F	GGGCCAGCATGTCAGGATTAGC	575	62
	4b-R	TGTGGGTGAGTGGATGGGAACG		
	RFLP-F	CGGGAGAAGCAGCCAAACTATG	237	65
	RFLP-R	CTTCTTCCTGGCCCGCTGTGAG		
*VSX2*	1-F	AGGGGGCACCTGGGACCAAC	586	72
	1-R	CCCGGCCTGGCAGGAACTTT		
	2-F	CGGCGCGGGAGAGGTCAG	427	66
	2-R	GCAGATTCCGCCAAACAAC		
	3-F	TGCCCAGGAGACACAGAGG	347	64
	3-R	ACATGATCATAGGCAAGCACA		
	4-F	AGGACGCCCTGCTGGAGAAA	380	68
	4-R	CCAAGTGCCCCTGCCTCAA		
	5-F	CTGCGGTGTGGGGAGTAAG	574	65
	5-R	GTGGGGAACAGGGAGGATG		
*CRYBA4*	2-F	ACTCCTGGACTCCCTATGTG	319	60
	2-R	ATTCAACCTCCCTGTATGTG		
	3-F	TCTTGCCTTCCTGGCTCCTG	430	62
	3-R	TGCGCAACCTGCATAATCTT		
	4-F	CCCCTGAATGGTTGTGACT	396	62
	4-R	AACCGAGGCTTGGAGAGGAA		
	5-F	AAGGGCAAATGGCAAGGTT	415	62
	5-R	TGGGCATCAGAGCACAAAAG		
*GDF6*	1-F	GGCGGGGCCGGGGTTTGT	635	72
	1-R	TAGCCTCCAGCGGGAACAGC		
	2a-F	CGGCCGACCTGCCCCCACTC	504	70
	2a-R	CCGGCCGAAGCCCAGACTCC		
	2b-F	GCCGGCCGGCTGGGAAGTCT	557	70
	2b-R	GCGAGCGCAGCGGGAAGTCG		
	2c-F	GCGCACGGCCTTCGCCAGTC	469	70
	2c-R	CCAGCGCCAGCTTCCTCCTC		
*OTX2*	3-F	TTTGCTTTGCCCTTAGTTCC	424	62
	3-R	CCCTGTTCTCTGCTTGGTCA		
	4-F	ACGGTGGGGAGAGCATTGGT	445	62
	4-R	CCTGGCCCCTTAGTGAGTGA		
	5a-F	CTGCCCATGTAGGATAGATT	440	61
	5a-R	ATGCCCCCAAAGTAGGAAGT		
	5b-F	GCTTCCATCTCCCCACTGTC	562	61
	5b-R	GGCCCTTCGTTTTTCCTTCT		
*RAX*	1-F	TTCGCCCGCGGAGCTTGACCT	527	66
	1-R	CCCCAACCCCGCGCCCAGTT		
	2-F	CCATCGCCGCCCTCACCA	522	66
	2-R	ACTCTGGGCATGCCAAGTCG		
	3-F	TTGAGGGGGACGGAGTGGAG	716	69
	3-R	GCAGGCGACAGGGAAAGAGG		
*SIX3*	1a-F	TCATCGCCCCTCTCCTCCTCTT	398	70
	1a-R	GCCGCTCGATGTCGCCCGTCTC		
	1b-F	CGGCGGCGGCGGCTCCAG	387	72
	1b-R	CGCACGCGGTACTTGTCCAC		
	1c-F	GCGTGCGAGGCCATCAACAA	552	65
	1c-R	CACGGCTTCCCTGGCTCTCA		
	2-F	GCTCGGGTTCTGCCTCTC	457	64
	2-R	TCGGTTTGTTCTGGGGATGG		
*SIX6*	1a-F	TGTGTCCCGCTGCCCCAATC	514	65
	1a-R	TTCTGTTCGCCGTCCCAAATG		
	1b-F	CCTTTCACGGTGGCAACTAC	514	65
	1b-R	GACAGACCGCGCTCCCAACTC		
	2-F	CGCCTTGCCGAGTAATCCT	447	70
	2-R	AGCCCGCGGGTCCCTGGTCAC		
	HA-SSCP-F	TCGCCTTAACTGCTGGGGTCTT	253	67
	HA-SSCP-R	AGTGGCCGCCTTGCTGGATA		
*SOX2*	1-F	CGCCTCCCCTCCTCCTCTC	443	68
	1-R	CGCCGGGGCCGGTATTTAT		
	2-F	GGGCGCCGAGTGGAAACTT	473	65
	2-R	GGGTGCCCTGCTGCGAGTA		
	3-F	CACGGCGCAGCGCAGATGC	459	65
	3-R	TTTGCACCCCTCCCATTTC		
*LRP6*	1-F	CTCCTCGCCTCCCCCACTTCTG	279	72
	1-R	CTGCTCCCGGGCCCCTTTCTCT		
	2-F	ATTTTCGACAGTCTTTGCTCAC	589	65
	2-R	TTCTTTTCTCATAGGGGTCAGG		
	3-F	GCGCGGCCTGAGCTTTCTTTA	410	64
	3-R	CTTCTTCCCCTCTGGCACTTAG		
	4-F	ATTTTAATGGGAGAGGTGACG	395	60
	4-R	TTTATTCCCGCCAACTATCTTT		
	5-F	AATTTTGGCTTATCACAGTT	330	58
	5-R	GGTCTCCCAAAGCAGTAT		
	6-F	TTTTATATTTATTTTTCAGTTC	575	50
	6-R	ATGTTATCTTAGTCAATGTTTT		
	7-F	GGGATGGATCTCACCTTTAG	478	58
	7-R	GATCAGCAGCCATTTCTCA		
	8-F	GGGGGAAAAGTGGTCAAA	538	56
	8-R	GGGGGCAGTAAAGAAGGT		
	9-F	TGGGAGCAAGACATAATCATAG	690	64
	9-R	TGGCACGCACCTGTAGTCCT		
	10-F	GGATCCTCTTGCCCCTGACA	560	62
	10-R	TAACCCATTCCCCTCTTTCTTC		
	11-F	ATTGTAGCCGTGATTTTGTTTA	577	58
	11-R	TCAGGAGTATCTAGGGAGTTAT		
	12-F	AAGCATGGGGTCAGAAGATAGA	777	62
	12-R	AAAGTGCTGGGTTACGGACATA		
	13-F	TGAGGGCATGCCAAAGAAT	499	58
	13-R	AATAAGCTACCAGGTCCAGAAT		
	14-F	GTGTGCCCATGTAGGTGTAAGC	511	64
	14-R	TAGTGGCCCAGGAAAGAAAGTC		
	15-F	CCGCCTCAGTCTCCCAAAGT	491	68
	15-R	TGCCAAGAAATGTGCCAAAAAC		
	16-F	TATCTAGTTTATTGGCTGTT	506	52
	16-R	CTAAAAGTGCATGAAAGTCT		
	17-F	AAGCTGATTATACATTTGATTT	403	64
	17-R	GGGCAGGGTGGCAGAGAA		
	18-F	TAAAGGAAGTAATGTGAAAACC	521	58
	18-R	TGAAAAACCCCAACTGAC		
	19-F	AGGCACCTTTTGATTCTTG	495	61
	19-R	CGCCCGGCTGATTTCTATGTAT		
	20-F	TTCAGGGCGTGGTATGTATGT	578	58
	20-R	TATCTAAGGCCTTCTGTGTAAA		
	21-F	AGCTATTCTTGGCCTTGTTCTA	508	61
	21-R	AGTCCTTTGAGCCTTTTATGC		
	22-F	TTTTAGCCATGATGAGGTCTTA	373	64
	22-R	GGGGCTATATCAGGTCCACAAC		
	23-F	GAAAATTGCCTCTTGGTCTGTG	550	65
	23-R	TGGTCTGCCTCATCCTTCTCTA		

### Heteroduplex-single strand conformation polymorphism analysis

The c.608G>A variation detected in *SIX6* was further evaluated in 96 normal controls by heteroduplex-single strand conformation polymorphism (HA-SSCP) analysis, as previously described [24], using an extra pair of primers (Table 2). Briefly, PCR products were mixed with an equal volume of formamide dye loading buffer. Then 1–4 μl of the mixture was loaded on 40 cm×30 cm×1 mm 8% polyacrylamide gels containing 10% glycerol. The DNA samples were separated by electrophoresis for 8–9 h at room temperature without temperature control. The DNA fragments were visualized by silver staining.

### Restriction fragment-length polymorphism analysis

The variation detected in *BMP4* c.751C>T was further evaluated in available family members, as well as in 96 normal controls, by restriction fragment-length polymorphism (RFLP) analysis using an extra pair of primers (Table 2). Since the c.751C>T variation in *BMP4* erased an enzyme recognition site of CviAII, wild amplicons were digested into four fragments (78, 76, 68, and 15 bp) while the variant amplicons were cut into three pieces (154, 68, and 15 bp).

## Results

### Mutation analysis

Eighteen nucleotide substitutions (Table 3), including two novel missense variations, eight known SNPs, and eight new synonymous changes, were detected upon complete sequencing analysis of the coding exons and the adjacent intronic regions of *BMP4*, *VSX2*, *CRYBA4*, *GDF6*, *OTX2*, *RAX*, *SIX3*, *SIX6*, *SOX2*, and *LRP6*. Of the two novel heterozygous missense variations, one was c.608G>A (p.R203Q) in *SIX6* and the other was c.751C>T (p.H251Y) in *BMP4*.

**Table 3:** Sequence variations found in *BMP4*, *CRYBA4*, *GDF6*, *LRP6*, *RAX*, SIX3, *SIX6*, *SOX2*, and *VSX2*.

**Gene**	**Exon**	**Sequence variation**	**Amino acid change**	**Patient number**	**Result**
BMP4	4	c.455T>C	S155S	TT:TC:CC = 19:10:3	rs17563
	4	c.751C>T	H251Y	1	Novel variation
CRYBA4	Intron 2	c.40-71C>T	No splice site change	2	rs2071860
	Intron 3	c.158+58C>T	No splice site change	3	rs58707060
	Intron 3	c.159-20A>G	No splice site change	AA:A/G:GG = 8:19:13	rs59023621
	Intron 5	c.444-18g>a	No splice site change	4	
GDF6	1	c.255G>T	P85P	1	
LRP6	5	c.867C>T	D289D	1	
	11	c.2450C>G	S817C	1	rs2302686
	14	c.3184G>A	V1062I	5	rs2302685
RAX	1	c.132C>A	D44E	6	rs2271733
	3	c.882A>G	Q294Q	4	
SIX3	1	c.90G>T	A30A	4	
SIX6	1	c.421C>A	N141K	7	rs33912345
	2	c.637C>T	P213S	1	
	2	c.608G>A	R203Q	1	Novel variation
SOX2	1	c.573A>G	A191A	1	
VSX2	4	c.750G>A	P250P	1	

No variation was identified in *OTX2*.

For internal quality control, the c.608G>A variation in *SIX6* was detected in an individual when her sample was used to optimize the experimental condition, but was not present in 96 unrelated normal controls. She was a three-month-old girl who had typical congenital aniridia with normal cornea size (a bilateral cornea diameter of 10 mm at the age of 3 months, within the normal range at this age) and a previously determined novel *PAX6* mutation (c.718C>T, p.R240X). This suggested that the c.608G>A variation in *SIX6* did not play additive effect and, therefore, might not be causative.

The c.751C>T variation in *BMP4* was detected in a proband suspected for microphthalmia (Figure 2), but was not detected in 96 unrelated normal controls. BMP4 alignment among six different species showed that the residue at 251 of BMP4 protein is highly conserved (Figure 2C). This ocular biometry measurement did not fully meet the criteria for micropthalmia, but did demonstrate an obviously small cornea and short axial length (Table 4). Besides this, he had bilateral corneal opacities, multiple pupils, an persistent iris membrane, and anterior pole cataract (Figure 2D-E). Unexpectedly, the c.751C>T variation was also present in his healthy brother with a normal ocular phenotype, including a normal anterior segment and normal axial length (Table 4 and Figure 2F-G). His sister (II:1 in Figure 2) and parents (I:1 and I:2 in Figure 2) were reported to be normal, but were unavailable to have ocular biometry. The variation was present in both the proband and in his healthy brother, and at least one of the parents (in whom only the proband had an abnormal ocular phenotype).

**Table 4:** Ocular biometry of the individuals with the *BMP4* mutation.

**Individual**	**Eye**	**Best visual acuity**	**Cornea diameter (mm)**	**Anterior chamber depth (mm)**	**Ocular length (mm)**
Proband	OD	0.5	10.7	2.25	20.28
	OS	0.3	10.8	2.23	20.13
Healthy brother	OD	1.5	11.4	3.39	22.78
	OS	1.5	11.6	3.32	22.74

Ocular biometry was measured by using IOL Master V5 (Carl Zeiss Meditec AG, Jena, Germany). The proband had a small cornea and short axial length. His brother had normal ocular biometry (normal range in Chinese adults: 12.12±0.40 mm for cornea diameter and 23.60±0.79 mm for axial length).

## Discussion

Normal development of the eye involves a complex process. Both genetic and environmental factors may play roles in the malformation of the eye. Although mutations in several genes have been detected in patients with microphthalmia or coloboma, such mutations are only detected in a small percentage of patients. In addition, these genes have not been analyzed simultaneously in any cohort of microphthalmia and/or coloboma cases.

 In the present study, ten genes previously reported to be responsible for microphthalmia and/or uveal coloboma were analyzed simultaneously in 32 Chinese patients with microphthalmia and/or uveal coloboma. Upon complete screening of the coding exons and adjacent intronic regions of *BMP4*, *VSX2*, *CRYBA4*, *GDF6*, *OTX2*, *RAX*, *SIX3*, *SIX6*, *SOX2*, and *LRP6*, no causative mutation was detected. This is the first systemic analysis of all ten genes in a series of microphthalmia and coloboma patients, and is the first analysis of most such genes in Chinese patients. The results suggest that the genetic cause of microphthalmia and uveal coloboma in Chinese is largely unknown.

*SIX6* encodes a nuclear homeoprotein and is expressed in the developing retina, optic stalk, and the hypothalamic and pituitary regions. Interstitial deletions at 14q22.3-q23, where *SIX6* is located, were found in three patients with bilateral anophthalmia, absence of the optic nerve, and chiasm and pituitary abnormalities [25]. Gallardo et al. [14] identified a heterozygous c.493A>G (p.T165A) variation in SIX6 in a patient with bilateral microphthalmia, cataract, and nystagmus from among a series of 73 patients with syndromic or nonsyndromic sporadic clinical anophthalmia and microphthalmia. However, this variation was also present in the healthy father, although it was not detected in more than 160 chromosomes from normal individuals. Aijaz et al. [26] did not find *SIX6* mutation 173 patients with microphthalmia, anophthalmia, and coloboma. In that study, the c.608G>A variation in *SIX6* was detected in an individual with typical congenital aniridia and a previously determined *PAX6* mutation (c.718C>T, p.R240X). Overall, there is no firm evidence that mutation in *SIX6* alone can cause microphthalmia, anophthalmia, or coloboma.

The *BMP4* gene product is a regulatory molecule functioning in mesoderm induction, tooth development, limb formation, bone induction, and fracture repair. *BMP4* is located in 14q22-q23, where recurrent interstitial deletions have been associated with anophthalmia-microphthalmia [9]. Bakrania et al. [9] identified a c.226del2 (p.S76fs104X) mutation at *BMP4* in a family whose carrier members had various phenotypes, including anophthalmia-microphthalmia, retinal dystrophy, myopia, brain anomalies, and polydactyly. In another family, a c.278A>G (p.E93G) mutation was found. However, these two mutations were also present in one of the phenotypically normal parents from each family [9]. On the other hand, three missense mutations in *BMP4* were reported in patients with orofacial cleft 11 (OMIM 600625) [27]. In that study, the c.751C>T variation in *BMP4* was present in a patient with microphthalmia, as well as in his healthy brother, and possibly in one of his normal parents. Therefore, further study is needed to reveal the role of *BMP4* in micropthalmia.

In summary, the c.608G>A variation in *SIX6* and the c.751C>T variation in *BMP4* might be reported as causative mutations if the *SIX6* c.608G>A variation is detected in a patient without a confirmed genetic basis, and if cosegregation analysis is not performed for the *BMP4* c.751C>T variation. Additional studies are expected to validate the association of microphthalmia and uveal coloboma with mutations in *SIX6* and *BMP4*. Great care is needed in making mutation–disease associations based on marginal evidence, especially for those genes with only a few identified mutations. Future genetic analyses of additional patients, as well as of other candidate genes, may enrich our understanding of the molecular basis of microphthalmia and uveal coloboma, as well as of the genotype–phenotype correlation.
